# The effect of ibrutinib on the myeloid cell compartment in CNS lymphoma

**DOI:** 10.1038/s41375-025-02600-y

**Published:** 2025-04-10

**Authors:** Julia C. Kuehn, Nicolas N. Neidert, Junyi Zhang, Jurik Mutter, Stefan Alig, Christian Klingler, Fabian Hummel, Lavanya Ranganathan, Sabine Bleul, Jürgen Beck, Marco Prinz, Maximilian Diehn, Ash Alizadeh, Justus Duyster, Roman Sankowski, Dieter H. Heiland, Florian Scherer

**Affiliations:** 1 Department of Medicine I, Medical Center-University of Freiburg, University of Freiburg, Freiburg, Germany; 2 Department of Neurosurgery, Medical Center-University of Freiburg, University of Freiburg, Freiburg, Germany; 3 Department of Medicine, Divisions of Oncology and Hematology, Stanford University, Stanford, CA USA; 4 Department of Hematology, Oncology and Stem Cell Transplantation, University Medical Center Essen, Essen, Germany; 5 Institute of Neuropathology, Medical Center-University of Freiburg, University of Freiburg, Freiburg, Germany; 6 BIOSS Centre for Biological Signalling Studies and Centre for Integrative Biological Signalling Studies (CIBSS), University of Freiburg, Freiburg, Germany; 7 Department of Radiation Oncology, Stanford University Medical Center, Stanford, CA USA; 8 German Cancer Consortium (DKTK) partner site Freiburg and German Cancer Research Center (DKFZ), Heidelberg, Germany; 9 Department of Neurosurgery, University Hospital Erlangen, Friedrich-Alexander University Erlangen-Nürnberg, Erlangen, Germany; 10 Department of Neurological Surgery, Lou and Jean Malnati Brain Tumor Institute, Robert H. Lurie Comprehensive Cancer Center, Feinberg School of Medicine, Northwestern University, Chicago, Illinois USA

**Keywords:** Translational research, Tumour immunology, Cancer microenvironment, Cancer models

The Bruton’s tyrosine kinase inhibitor (BTKi) ibrutinib effectively blocks downstream B-cell receptor activation and has demonstrated clinical efficacy both as monotherapy and in combination with immunochemotherapy in patients with central nervous system lymphoma (CNSL) [1, 2]. Beyond its direct effect on lymphoma cells, ibrutinib regulates the tumor microenvironment (TME) and enhances T-cell immunity and function in systemic B-cell Non-Hodgkin lymphomas (NHL) such as chronic lymphocytic leukemia (CLL) [3, 4]. However, its impact on the tumor immune landscape of B-cell lymphomas within immune-privileged sites like the CNS remains largely unexplored, primarily due to challenges associated with the availability of appropriate CNS lymphoma tissue following ibrutinib treatment.

In this study, we used human slice cultures from a CNSL patient undergoing complete brain tumor resection due to a suspected glioblastoma to investigate the influence of ibrutinib treatment on the lymphoma immune microenvironment, particularly focusing on the myeloid cell compartment. We performed comprehensive genetic and transcriptional profiling as well as single nucleus RNA sequencing (snRNA-Seq) of the bulk FFPE tumor and CNSL slice cultures, which were processed according to a previously established approach and subjected to treatment over five consecutive days either with ibrutinib solved in DMSO (‘ibrutinib-treated’), DMSO alone (‘DMSO control’), or remained untreated (‘untreated’) (Supplementary Fig. 1, Supplementary Methods) [5]. Genetic analyses of the bulk tumor by targeted deep sequencing and shallow whole genome sequencing revealed the presence of CNSL-specific single nucleotide variants (SNVs) and copy number aberrations (CNAs), including mutations in *MYD88, CD79B, PIM1*, *OSBPL10*, and *TBL1XR1* genes (mean allele frequency [AF] = 38%) as well as gains of 9p24 (*PD-L1*) and losses of 6p21 (*HLA-D*) or 1p13 (*CD58*), which have been associated with immune evasion in CNSL [6] (Fig. 1A, Supplementary Tables 1, 2). The tumor was classified as an activated B-cell like (ABC) diffuse large B-cell lymphoma (DLBCL) and assigned to the MCD subtype and LE1 ecotype, which both are typically associated with an ABC cell-of-origin [7–9] (Supplementary Table 3). Clonal rearrangements of immunoglobulin genes were predicted to be *IGHV4-59* for the heavy chain and *IGKV3-29* for the light chain [10]. Importantly, the majority of tumor-intrinsic genetic aberrations were also present in DNA isolated from the slice cultures, confirming the presence of the same malignant B-cell clone in the individual culture conditions, with mean AFs ranging from 1.05% in the ibrutinib-treated to 4.96% in the untreated slices (Fig. 1A, Supplementary Tables 1, 2). We generated pseudo-H&E images from tumor sections using Stimulated Raman Histology, demonstrating that a small proportion of the slide was infiltrated with lymphoma cells, which mirrors the low mutant AFs detected by slice culture DNA sequencing in comparison to the bulk tumor **(**Fig. 1B**)**.

**Fig. 1:**
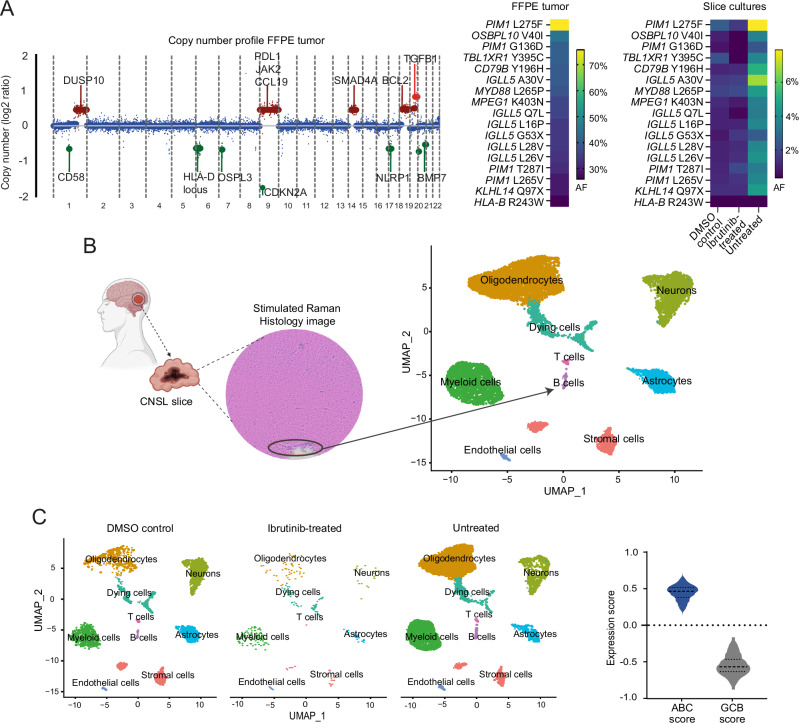
CNSL tumor and slice culture characterization. **A** Copy number (left) and mutational (right) profile of the FFPE-embedded tumor and tumor slices treated with three different conditions. Selected genes in chromosomal regions with losses (green) or gains (red) are annotated. For the heatmaps, only missense and nonsense mutations were considered, the full genetic profile is listed in the Supplementary Table 1. AF, allele frequency; FFPE, formalin fixed paraffin embedded; DMSO, dimethyl sulfoxide. **B** Stimulated Raman Histology image of the tumor slice (left). The malignant B-cell proportion is highlighted by a black circle. Further, a UMAP of the integrated snRNA-Seq data of all tumor slices is shown (right). CNSL, central nervous system lymphoma; UMAP, uniform manifold approximation and projection. **C** UMAP of the snRNA-Seq dataset across each individual slice culture condition, colored by the annotated cell clusters. Further, a violin plot of the ABC (activated B-cell) / GCB (germinal center B-cell) expression scores obtained by bulkRNA sequencing across all cells in the B-cell cluster is shown. Dashed line, Median; Dotted line, 25–75th percentile within each violin plot. DMSO dimethyl sulfoxide, UMAP uniform manifold approximation and projection.

Next, we performed snRNA-Seq from slices of all three conditions (Supplementary Fig. 1, Supplementary Methods). In total, 12,173 nuclei passed quality control and were further analyzed by data integration to identify the presence of distinct cell types (Fig. 1B, Supplementary Table 4, Supplementary Fig. 2A–C, Supplementary Methods). Nine major cell types could be distinguished, with oligodendrocytes (34%), neurons (19.8%), and myeloid cells (16.3%) representing the most abundant cell populations, while B-cells were present in 1.2% of the total cell pool, mirroring the fractional abundance of malignant genetic aberrations in the slice cultures (Fig. 1B, Supplementary Fig. 2C). Importantly, this cell composition was largely maintained in all individual culture conditions (Fig. 1C, Supplementary Fig. 2C); yet, the ibrutinib-treated slices revealed no B-cells by snRNA-Seq despite the detection of lymphoma-specific mutations by targeted DNA sequencing (Fig. 1A, C, Supplementary Fig. 2C). B-cells from the integrated dataset showed high expression of genes associated with the ABC DLBCL subtype, consistent with the results of the Hans algorithm (Fig. 1C).

The myeloid cell compartment has emerged as a crucial population for modulating tumor immune responses in other brain cancer types and constitutes a significant component in our snRNA-Seq analyses [11]. Therefore, we next focused on the characterization of the myeloid cells to explore the specific effect of ibrutinib treatment on this population in CNSL. Five distinct clusters (C1-C5) within the myeloid compartment were defined based on expression profiles (Fig. 2A, Supplementary Fig. 3A–C, Supplementary Table 5). Importantly, the representation of these clusters was significantly different in ibrutinib-treated slices compared to the two control conditions, with markedly increased antigen-presenting (cluster C2) and decreased SPP-1-expressing cell populations (cluster C1, Fig. 2A, Supplementary Fig. 3D). Pseudotime analysis of the integrated myeloid cell compartment revealed two differentiation branches, one leading to SPP1-expressing cells (C1) and the other to the antigen-presenting cell state (C2, Fig. 2B). Expression level comparisons of SPP1 and genes that represent the antigen-presenting subpopulation (CIITA, HLA-DMB) showed significant differences between the ibrutinib-treated slices and slices of the control conditions (Fig. 2C, Supplementary Table 5). Collectively, these data indicate a modulatory effect of ibrutinib on the myeloid cell compartment in CNSL, shifting cells from an SPP1-expressing immunosuppressive phenotype to an immune-stimulating state.

**Fig. 2:**
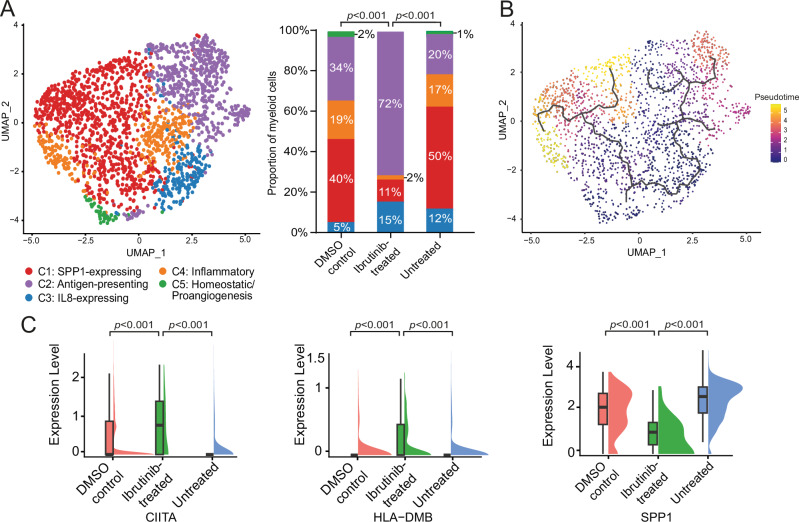
Ibrutinib induced changes in the myeloid compartment of CNSL. **A** UMAP showing the five different subclusters (C1–C5) identified within the myeloid cluster of the CNSL tumor slices (integrated dataset). The proportion of cells per subcluster among the total number of myeloid cells for each treatment condition is shown as a bar graph. UMAP, uniform manifold approximation and projection; DMSO, dimethyl sulfoxide. **B** UMAP of the myeloid compartment colored by pseudotime. Pseudotime trajectories are shown as black lines. UMAP, uniform manifold approximation and projection. **C** Rainbow plots of marker genes of the antigen-presenting myeloid subcluster (CIITA, HLA-DMB) and the SPP1 subcluster comparing the pseudo-bulk expression level of these genes among the three treatment conditions. Each plot shows the median, 25–75th percentile, and range of expression levels. DMSO, dimethyl sulfoxide.

To validate our findings, we collected cerebrospinal fluid (CSF) from a CNSL patient with leptomeningeal infiltration experiencing disease progression after ibrutinib monotherapy (Supplementary Fig. 4A, Supplementary Table 6). We applied snRNA-Seq to 1,995 cells isolated from CSF and identified three distinct clusters, with myeloid cells representing 0.7% of the total cell pool (Supplementary Fig. 4B, C). These myeloid cells expressed high levels of CIITA and HLA-DMB, while SPP1 expression remained low, reflecting the phenomena observed in the CNSL slice cultures treated with ibrutinib (Supplementary Fig. 4D). As a separate validation experiment, we further cultured non-malignant cortical slices from a patient undergoing epilepsy surgery under the same three treatment conditions and applied snRNA-Seq to a total of 4,134 cells (Supplementary Fig. 5A–C). Quantification of myeloid cell subclusters revealed a reduction of SPP1-expressing cells in cluster C1 and increase of cells with an antigen-presenting phenotype (C2) in ibrutinib-treated slices, aligning with the results from the CNSL slice culture experiments (Supplementary Fig. 5D). However, the effect appeared rather moderate, suggesting an underlying lymphoma-independent ibrutinib effect on the myeloid compartment that might be enhanced by lymphoma infiltration (Supplementary Fig. 5D, E).

In conclusion, we here present a single slice culture model analysis demonstrating a modulatory effect of ibrutinib on the myeloid cell compartment of the brain, redirecting SPP1-expressing myeloid cells, known for their largely immunosuppressive properties, to an antigen-presenting phenotype that is commonly associated with immune-stimulatory characteristics [12, 13]. To the best of our knowledge, this is the first report of an analysis investigating human CNSL tissue and its TME following BTKi therapy. In CLL, various research studies have explored the role of ibrutinib on the immune environment, describing an enhanced T-cell immunity and improved cytotoxic T-cell response as a consequence of irreversible *inducible T-cell kinase* (ITK) inhibition, contributing to its therapeutic effect in this B-cell lymphoma subtype [3, 4]. The immune landscape of CNSL differs remarkably from extracerebral B-cell lymphoma entities due to its localization within an immune-privileged organ [14]. Yet, obtaining tissue samples suitable for comprehensive TME profiling in CNSL represents a considerable challenge, as repeated biopsies are typically not performed in relapse situations in clinical practice, and moreover, stereotactic biopsies generally yield only minute tumor fragments, which are usually insufficient for extensive TME analyses. Therefore, patient-derived tumor slice cultures presented a unique opportunity to explore the CNSL TME longitudinally in response to ibrutinib treatment in this study. Warranting further validation, our observations might have therapeutic implications for CNSL patients. The findings suggest a biological mechanism that supports the concomitant use of ibrutinib in the context of CAR T-cell therapies through the engagement of an immunostimulatory myeloid TME in CNSL [13, 15]. In fact, clinical trials assessing the efficacy of CAR T-cells in PCNSL encourage continuation of ibrutinib treatment up until 3 months after CAR T-cell infusion [15]. Although the results presented here are promising, our study is inherently limited by this single-case analysis and culture conditions that might not fully capture the complexity of human brain processes. Therefore, it should be interpreted with caution, and further validation experiments in xenograft mouse models or human tissue obtained after BTKi treatment are needed to substantiate our findings and overcome the limitations associated with this solitary observation. Moreover, the scarcity of T-cell subpopulations within the individual slice culture conditions precluded comprehensive exploration of the T-cell compartment and its interactions with myeloid cells, omitting an important component of the CNSL TME from our analysis.

In summary, based on a unique human slice culture model from primary CNSL tissue, our data suggest a modulating effect of ibrutinib on the myeloid compartment in human CNSL by shifting myeloid cells from an immunosuppressive phenotype expressing SPP1 to an antigen-presenting cell state, indicating an unknown immune-activating effect of ibrutinib on the brain TME with potential further implications on combinatory treatment strategies involving ibrutinib together with CAR T-cell therapies.

## Supplementary information

Data Supplement

Supplementary Tables

## Data Availability

Tumor mutational data and other relevant data are provided in the Supplementary Data. Owing to restrictions related to the dissemination of germline sequence information included in the informed consent forms used to enroll study subjects, we are unable to provide access to raw sequencing data. Reasonable requests for additional data will be reviewed by the authors to determine whether they can be fulfilled in accordance with these privacy restrictions.
