# Supplementary material for: The effect of ibrutinib on the myeloid cell compartment in CNS lymphoma
Source: Leukemia. 2025 Apr 10;39(6):1532–5. doi: 10.1038/s41375-025-02600-y (PMC12133588; doi:10.1038/s41375-025-02600-y)
Supplement: Supplementary file 1 — Data Supplement [file 41375_2025_2600_MOESM1_ESM.pdf]

Data Supplement for

The effect of ibrutinib on the myeloid cell compartment in CNS lymphoma

Julia C. Kuehn, Nicolas N. Neidert, Junyi Zhang, Jurik Mutter, Stefan Alig, Christian Klingler, Fabian Hummel, Lavanya Ranganathan, Sabine Bleul, Jürgen Beck, Marco Prinz, Maximilian Diehn, Ash Alizadeh, Justus Duyster, Roman Sankowski, Dieter H. Heiland, Florian Scherer^

^ Corresponding author. Email: florian.scherer@uniklinik-freiburg.de

<u>**Table of contents**</u>

Supplementary Methods

Supplementary Figures

Supplementary Tables – attached separately

Supplementary Table 1. SNVs detected in the FFPE tumor tissue and treated tumor slices.

Supplementary Table 2. CNAs identified in the FFPE tumor tissue that were also detected in tumor slices.

Supplementary Table 3. Clinicopathological characteristics of the tumor.

Supplementary Table 4. Number of nuclei analyzed per treatment condition.

Supplementary Table 5. Top 50 differentially expressed genes per myeloid cluster.

Supplementary Table 6. SNVs detected in the CSF of a CNSL patient treated with ibrutinib.

Supplementary Methods

Patients and tissue collection

Biospecimens were obtained from three different patients treated at the Department of Hematology and Oncology as well as the Department of Neurosurgery of the University Medical Center Freiburg, Germany. Patients provided written informed consent for the collection of specimens and experiments performed in this study, in accordance with the declaration of Helsinki and approved by the local ethics committee (DRKS00015307, 23-1234-S1, 23-1233-S1). 1) Tumor tissue was obtained from a patient undergoing surgical resection of a left temporal brain lesion due to a suspected glioblastoma. The patient received dexamethasone before surgical resection. The tumor was histopathologically classified as large B-cell lymphoma of immune-privileged sites according to the current 2022 WHO classification (EBV-negative), revealing an activated B-cell (ABC) like subtype by the Hans algorithm (1,2). 2) CSF was obtained from a patient with an EBV-negative secondary central nervous system lymphoma (CNSL) with leptomeningeal infiltration undergoing ibrutinib treatment. 3) Cortical tissue was obtained from a patient undergoing epilepsy surgery.

Tissue slice culture and treatment

Human neocortical tissue cultures from the CNSL patient 1) and the cortical tissue of patient 3) were prepared as described in Ravi et al. (3,4). Briefly, neocortical tissue was collected in Hibernate medium supplemented with 13 mM Glucose and 30 mM NMDG. 300µm-thick coronal tissue sections were prepared using a vibratome (VT1200, Leica Biosystems, Nussloch, Germany) and incubated in preparation medium for 10 min before plating. The first slice was imaged using Stimulated Raman Histology (NIO®Laser Imaging System, INVENIO, Santa Clara, CA, USA) (5). Two to

three sections per well were cultured in a growth medium containing Neurobasal (l-glutamine) supplemented with 2% serum-free B-27, 2% Anti-Anti, 10 mM Glucose, 1 mM MgSO4, and 1 mM Glutamax, at 5% CO2 and 37 °C. Culture medium was replaced 24 h post-plating, and every 48 h thereafter.

After 5 days, 5 to 6 slices were treated/incubated under the following conditions: i) with culture medium containing 1 ng/mL ibrutinib solved in DMSO (Dimethylsulfoxid, DMSO concentration: 0.03%), which represents physiological ibrutinib concentrations observed in the CSF of lymphoma patients ('ibrutinib-treated') (6–8), ii) culture medium containing 0.03% DMSO only ('DMSO control'), or iii) culture medium without any supplements ('untreated'). Conditions ii) and iii) served as controls. Treatment was performed every 24 hours for a total of 5 days. After 5 days, the slices were snap frozen and stored in liquid nitrogen for 4 to 10 weeks.

Single nucleus preparation of cultured slices and 10x Genomics droplet-based single-nucleus library preparation and sequencing

Tissue slices of each condition were thawed on ice. Pre-cooled EZ Lysis Buffer was added and the tissue was homogenized using a micro-pestle. After incubation for 5 min, the homogenate was centrifuged at 500 g for 4 min. The pellet was resuspended in EZ Lysis Buffer and incubated for another 5 min. After centrifugation at 500 g for 4 min, the supernatant was discarded and nuclei were resuspended in 500 μl pre-cooled Nuclei Suspension Buffer (1% BSA, 0.2 U/μl RNAse inhibitor, 1:1000 DAPI). After filtration through a 70μm Flowmi Strainer, the nuclei suspension was mixed with 30% Sucrose and layered on top of 500 μl 30% Sucrose. The gradient was centrifuged at 13 000 g for 30 min at 4°C. The myelin and debris layers were removed, and the pellet was resuspended in Nuclei Suspension Buffer. The suspension was sorted for 30 000

DAPI-positive events using a Beckman Coulter 14-color MoFlo Astrios cell sorter (Beckmann Coulter, Brea, CA, USA). After centrifugation at 300 g for 10 min at 4°C, nuclei were resuspended and loaded onto a Chromium Controller (10x Genomics, Pleasanton, CA, USA) using the Chromium Next GEM Single Cell 3' Reagent Kit v3.1. Library construction and sample indexing was performed according to the manufacturer's instructions. Each library was sequenced using a P2 Flow Cell (100 cycles) on the Illumina NextSeq1000 platform (Illumina, San Diego, CA, USA).

Single cell suspension and sequencing of CSF cells

CSF from patient 2) was frozen and thawed as described by Touil et al (9). Cells were loaded onto a Chromium Controller (10x Genomics) using the Chromium Next GEM Single Cell 3' Reagent Kit v3.1 and processed as described above.

Integration and analysis of the 10x single-nucleus transcriptome data

Transcriptome alignment to a human reference (GRCh38-2020-A) was performed using the CellRanger v.7.2.0 workflow. Analysis was performed using the Seurat 4.4 package. Cells with >10% mitochondrial transcripts, >7 500 or <200 features per cell were excluded from further analysis. Doublet removal was performed using the DoubletFinder 2.0 R package. All datasets were integrated using Harmony 1.2 (10). Annotation of major cell clusters was performed using SingleR with the celldex HumanPrimaryCellAtlasData dataset (11,12).

Additionally, known marker genes were used to perform manual cell annotation. Cells classified as dying cells showed a signature with enrichment of mitochondrial genes as well as genes associated with apoptosis.

Classification of cell-of-origin (COO)

The lymphoma COO of the FFPE tumor was determined by immunohistochemistry according to the Hans classifier (1). Further, we calculated ABC and GCB scores based on the snRNA-Seq dataset as described by Reddy et al. (13). Briefly, the average expression of 9 GCB and 11 ABC-associated genes was calculated among all cells classified as B-cells in the integrated snRNA-Seq dataset using the AverageExpression() function of the Seurat package (14) . The GCB/ABC score was calculated averaging the Z-normalized expression values.

Analysis of the myeloid compartment

Cells classified as myeloid cells in the integrated snRNA-Seq dataset were extracted and clustered into five groups (C1-C5) using the Seurat package in an unsupervised fashion. Using FindAllMarker(), differentially expressed genes for each cluster were identified (Top 50 listed in **Supplementary Table 5,** top 20 depicted in **Supplementary Fig. 3B**). Cells with a dendritic cell signature could not be identified in the dataset. Classical monocyte markers such as CD14 were only detectable in a minute subset of cells that were distributed across all five clusters, which might be explained by cultivation times exceeding the lifetime of monocytes (15) (**Supplementary Fig. 3A**). Differentiation between microglia and macrophages was complex in this context, as microglia-specific genes are likely downregulated under culture conditions, as described before (16). Due to these challenges, the annotation of clusters C1-C5 was based on the expression profiles of the myeloid cells per cluster, not on individual cell types (**Supplementary Fig. 3A,B**).The Monocle 3 package was used to order cells by pseudotime using the standard workflow. The cluster C3, mainly defined by IL8 expression, was defined as the root (17). The Ucell package was used to apply those

myeloid cluster signatures that were defined in the CNSL infiltrated slice cultures to the non-malignant cortical slice datasets (18).

DNA and RNA isolation

DNA and RNA were isolated from five 10μm sections of FFPE-embedded bulk tumor tissue using the AllPrep DNA/RNA FFPE Kit (QIAGEN, Venlo, Netherlands) according to manufacturer's instructions. DNA yield was 2 388 ng and RNA yield was 4 320 ng. From the nuclear suspensions of the individual slice conditions, DNA was isolated using the QIAamp DNA mini kit (QIAGEN). DNA yields were 28 ng for the ibrutinib-treated slices, 41 ng for the DMSO-treated slices, and 38 ng for the untreated slices. DNA from CSF was isolated as described previously (19). Briefly, fresh CSF was centrifuged at 500 g for 5 min. DNA isolations from supernatant and the cell pellet were performed individually using the QIAamp Circulating Nucleic Acid Kit (QIAGEN) and QIAamp DNA mini kit (QIAGEN) according to the manufacturer's protocol. The genomic DNA isolated from the CSF pellet was further sonicated using the Covaris system as described previously (1). The DNA yield of pooled supernatant cfDNA and sonicated genomic DNA from the pelelt was 225 ng.

bulkRNA sequencing

FFPE tumor RNA was used for bulkRNA sequencing. Ribosomal RNA was depleted using the NEBNext rRNA Depletion Kit v2 (New England Biolabs, Ipswich, MA, USA). Library preparation was performed using the NEBNext Ultra II Directional RNA Library Prep Kit according to manufacturer's instructions. Libraries were sequenced using a P2 Flow Cell (100 cycles) on the Illumina NextSeq1000 platform. Data were processed

using cutadaptor (20). Alignment using STAR was performed (21). Count data were generated using featureCounts (22). Data were normalized using the Deseq2 tool (23). The ecotype of the tumor was assessed using the Lymphoma EcoTyper (24). MiXCR was used to determine the clonotype of the tumor ('mixcr analyze rna-seq') (25).

CAPP-Seq and shallow whole genome sequencing (sWGS)

Cancer Personalized Profiling by Deep Sequencing (CAPP-Seq) was performed for mutational profiling of DNA of the FFPE-embedded tumor as well as DNA of the slice culture conditions, using a CNSL-specific custom sequencing panel as previously described (19). Sequencing was performed on the Illumina NextSeq1000 platform. Tumor variants identified in the bulk tumor by a custom genotyping pipeline and those mutations were monitored in the tumor slices using a custom monitoring MonteCarlo approach as previously described (19). Sequencing depth for the bulk tumor was 805X, for the ibrutinib-treated slices 808X, the DMSO control slices 1 020X, and the untreated control 728X. Genetic profiles of the tumor were used to classify them based on the LymphGen classifier (26).

Libraries generated by the CAPP-Seq workflow were also used for sWGS. Sequencing was performed in multiplexes of 64 libraries using the Illumina NovaSeq 6000 platform with an SP flow cell (300 cycles). Raw sequencing data was processed using a custom pipeline to generate sorted BAM files and WIG files segmented into 50 and 500 kilobase pairs (kbp). Genome-wide copy number aberration (CNA) analysis was conducted for bin sizes of 50 and 500 kbp using two tools optimized for low-depth sequencing: ichorCNA and Absolute Copy Number Estimation (ACE) (27,28). ACE was run directly from the sorted BAM files, assuming a diploid genome, with no additional modifications to the default settings. ichorCNA was run using the

corresponding WIG files, applying default parameters. A Panel of Normals (PoN), generated from 70 germline samples from patients with PCNSL, was used to enhance detection accuracy. These germline samples were sequenced using the same method described above. ichorCNA was used for CAN genotyping of the bulk tumor, ACE was utilized for CNA monitoring in slice cultures, as ACE revealed more robust results when allele frequencies were low.

Statistical analyses

Continuous variables were presented as median, 25th-75th percentile, and range (described in the figure legends) and compared using Student's t-test. Categorial variables were compared using X^2 test. *P*-values <0.05 were considered as significant. Statistical tests were performed using GraphPad Prism (version 9.1.1).

Supplementary Figures

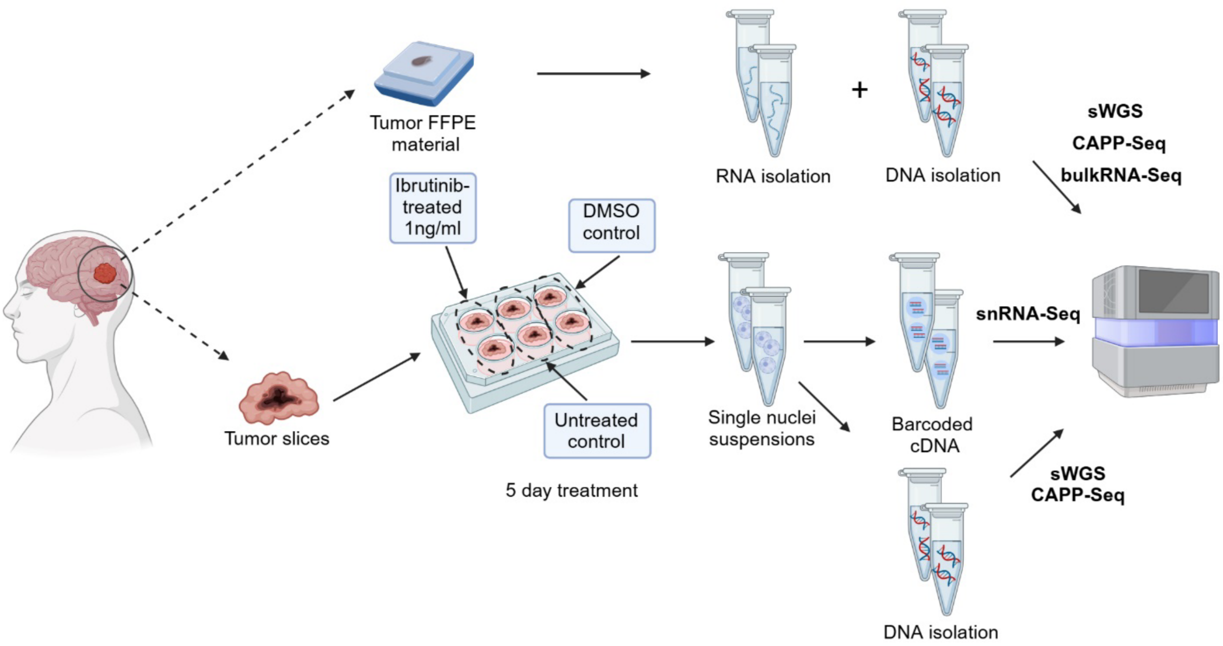

Supplementary Figure 1. Workflow of bulk tumor and slice cultures of the CNSL patient. Shallow whole genome sequencing (sWGS), targeted next generation sequencing (CAPP-Seq) and bulk RNA sequencing were performed on the FFPE-embedded tumor tissue. Slices of the CNSL tissue were cultured and treated over five days either with ibrutinib solved in DMSO, with DMSO alone, or they remained untreated. Single nuclei suspensions from these slice cultures were analyzed by single nucleus RNA sequencing (snRNAseq) as well as sWGS and CAPP-Seq. CAPP-Seq; Cancer Personalized Profiling by Deep Sequencing. bulkRNA-Seq; bulk RNA tumor sequencing.

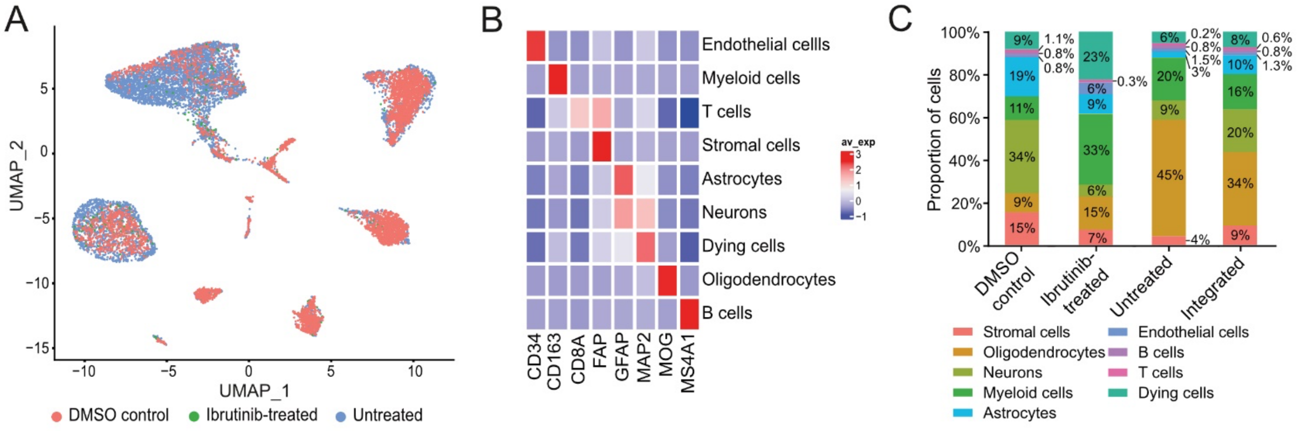

Supplementary Figure 2. Single nucleus RNA sequencing data from treated CNSL slices. (A) UMAP of the integrated snRNA-Seq dataset. Cells are colored by condition. **(B)** Heatmap of marker gene expression scores across annotated clusters. Each column represents a cell type specific marker gene, each row represents an annotated cluster. **(C)** Bar graph showing the proportion of cell types of the total number of cells for each treatment condition.

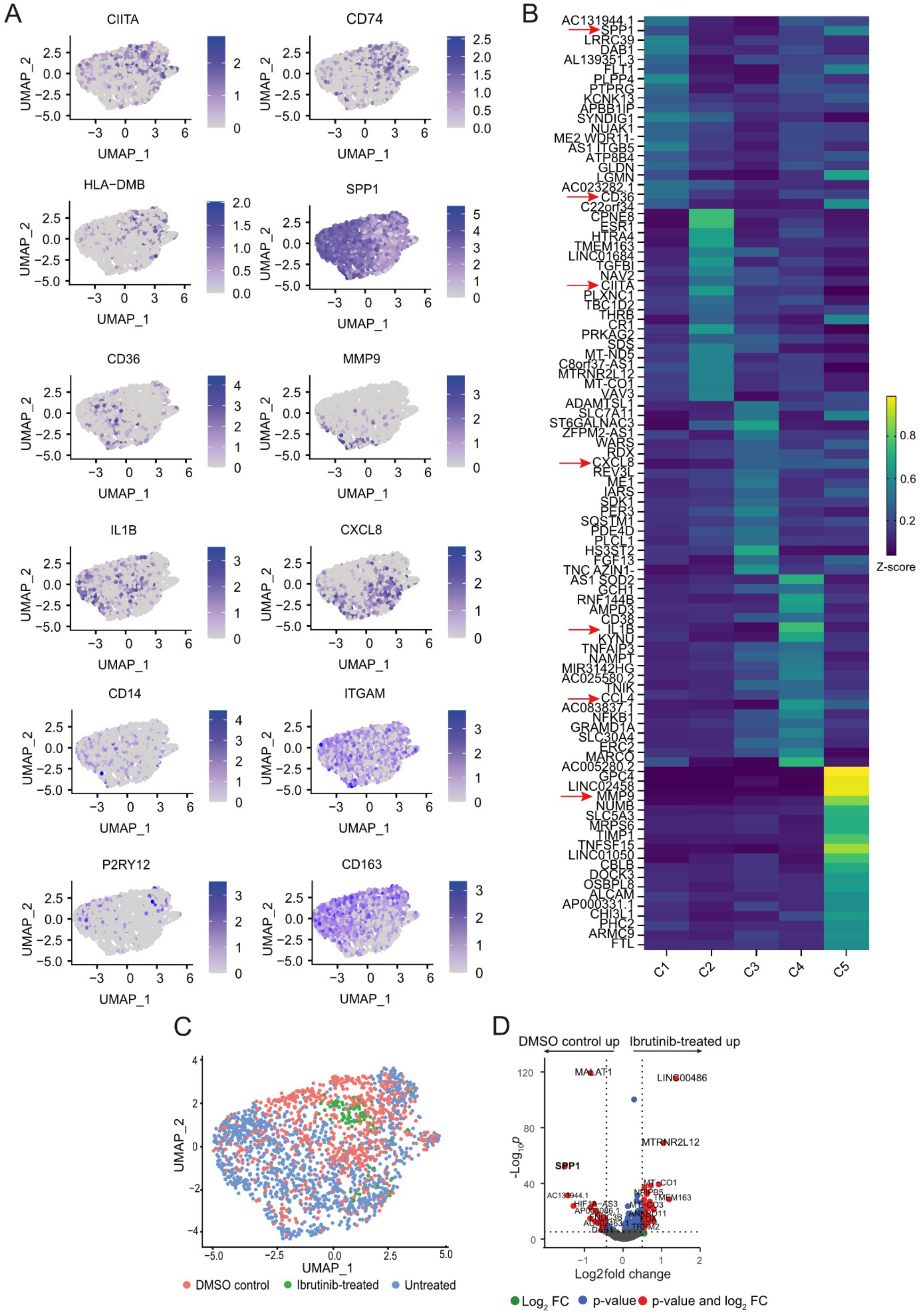
A
CIITA
CD74
HLA−DMB
SPP1
CD36
MMP9
IL1B
CXCL8
CD14
ITGAM
P2RY12
CD163
UMAP_1
UMAP_2
B
AC131944.1
SPP1
LRRC39
DAB1
AL139351.3
FLT1
PLPP4
PTPRG
KCNK13
APBB1IP
SYNDIG1
NUAK1
ME2 WDR11-
AS1 ITGB5
ATP8B4
GLDN
LGMN
AC023282.1
CD36
C22orf34
CPNE8
ESR1
HTRA4
TMEM163
LINC01684
TGFBI
NAV2
CIITA
PLXNC1
TBC1D2
THRB
CR1
PRKAG2
SDS
MT-ND5
C8orf37-AS1
MTRNR2L12
MT-CO1
VAV3
ADAMTSL1
SLC7A11
ST6GALNAC3
ZFPM2-AS1
WARS
RDX
CXCL8
REV3L
ME1
IARS
SDK1
PER3
SQSTM1
PDE4D
PLCL1
HS3ST2
FGF13
TNC AZIN1-
AS1 SOD2
GCH1
RNF144B
AMPD3
CD38
IL1B
KYNU
TNFAIP3
NAMPT
MIR3142HG
AC025580.2
TNIK
CCL4
AC083837.1
NFKB1
GRAMD1A
SLC30A4
ERC2
MARCO
AC005280.2
GPC4
LINC02458
MMP9
NUMB
SLC5A3
MRPS6
TIMP1
TNFSF15
LINC01050
CBLB
DOCK3
OSBPL8
ALCAM
AP000331.1
CHI3L1
PHC2
ARMC9
FTL
C1
C2
C3
C4
C5
Z-score
C
UMAP_1
UMAP_2
DMSO control
Ibrutinib-treated
Untreated
D
DMSO control up
Ibrutinib-treated up
MALAT1
LINC00486
MTRNR2L12
SPP1
MT-CO1
TMEM163
AC131944.1
Log2fold change
$-Log_{10}P$
Log₂ FC
p-value
p-value and log₂ FC

Supplementary Figure 3. Myeloid compartment of the CNSL slices. (A) Feature plot of 8 different marker genes defining the five subclusters of the myeloid compartment and 4 cell type specific marker genes of the snRNA-Seq dataset. UMAPs are colored in blue based on the expression level of each gene. **(B)** Heatmap of expression level Z-scores of the top 20 differentially expressed genes per cluster (C1-C5). Genes of special interest are marked with arrows. **(C)** UMAP of the myeloid cluster of the integrated snRNA-Seq dataset. Cells are colored by condition. **(D)** Volcano plot showing differentially expressed genes of a pseudo-bulk analysis of the ibrutinib-treated CNSL slices compared to the DMSO control CNSL slices. The *p*-value cut-off is 0.05 and the log2 fold change (Log2 FC) cut-off is 0.5. The top 15 differentially expressed genes are annotated.

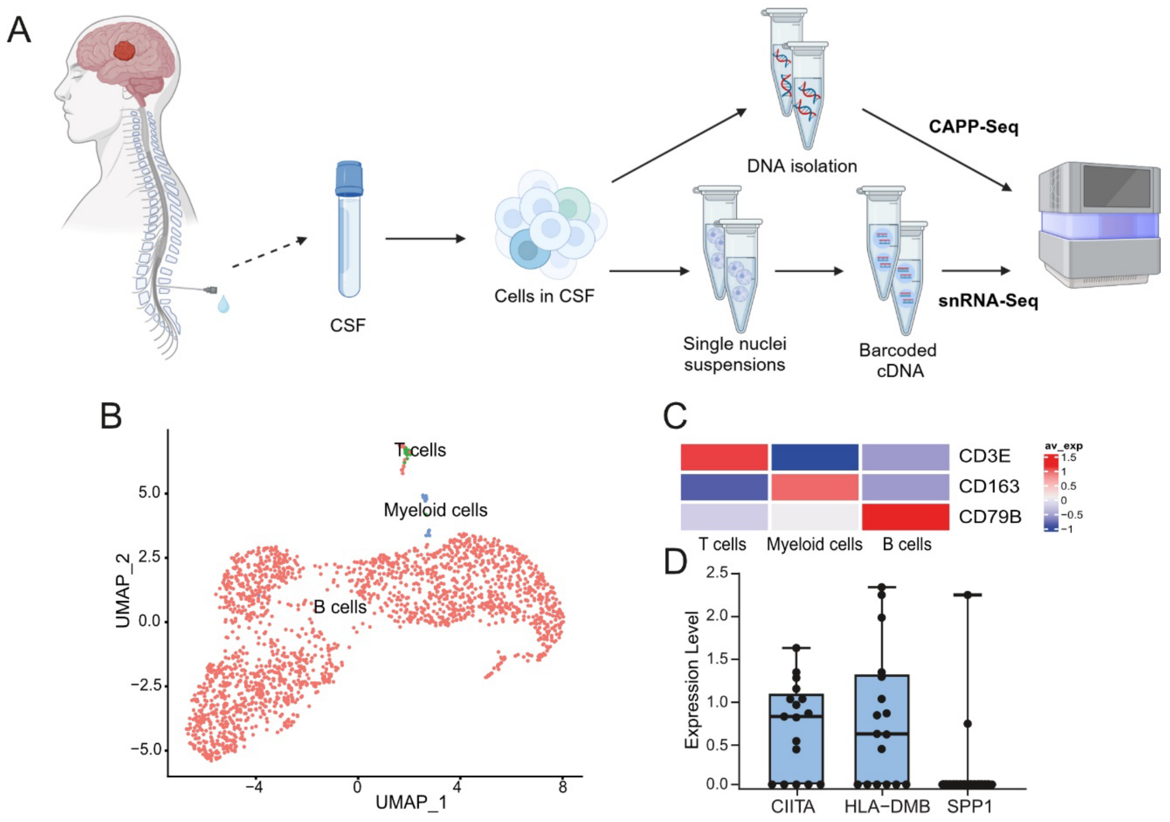

Supplementary Figure 4. Single cell RNA sequencing dataset of CSF cells from a CNSL patient treated with ibrutinib. (A) Cells isolated from CSF of a CNSL patient with leptomeningeal lymphoma infiltration treated with ibrutinib monotherapy were used for targeted sequencing by CAPP-Seq as well as snRNA-Seq single. (B) UMAP of the single cell datasets colored by annotated clusters. **(C)** Heatmap of marker gene expression levels across annotated clusters. Each column represents an annotated cluster, each row represents a cell type specific marker gene. **(D)** Box plot showing the expression levels of CIITA, HLA-DMB and SPP1. Each plot shows the median, 25-75th percentile, and range of expression levels.

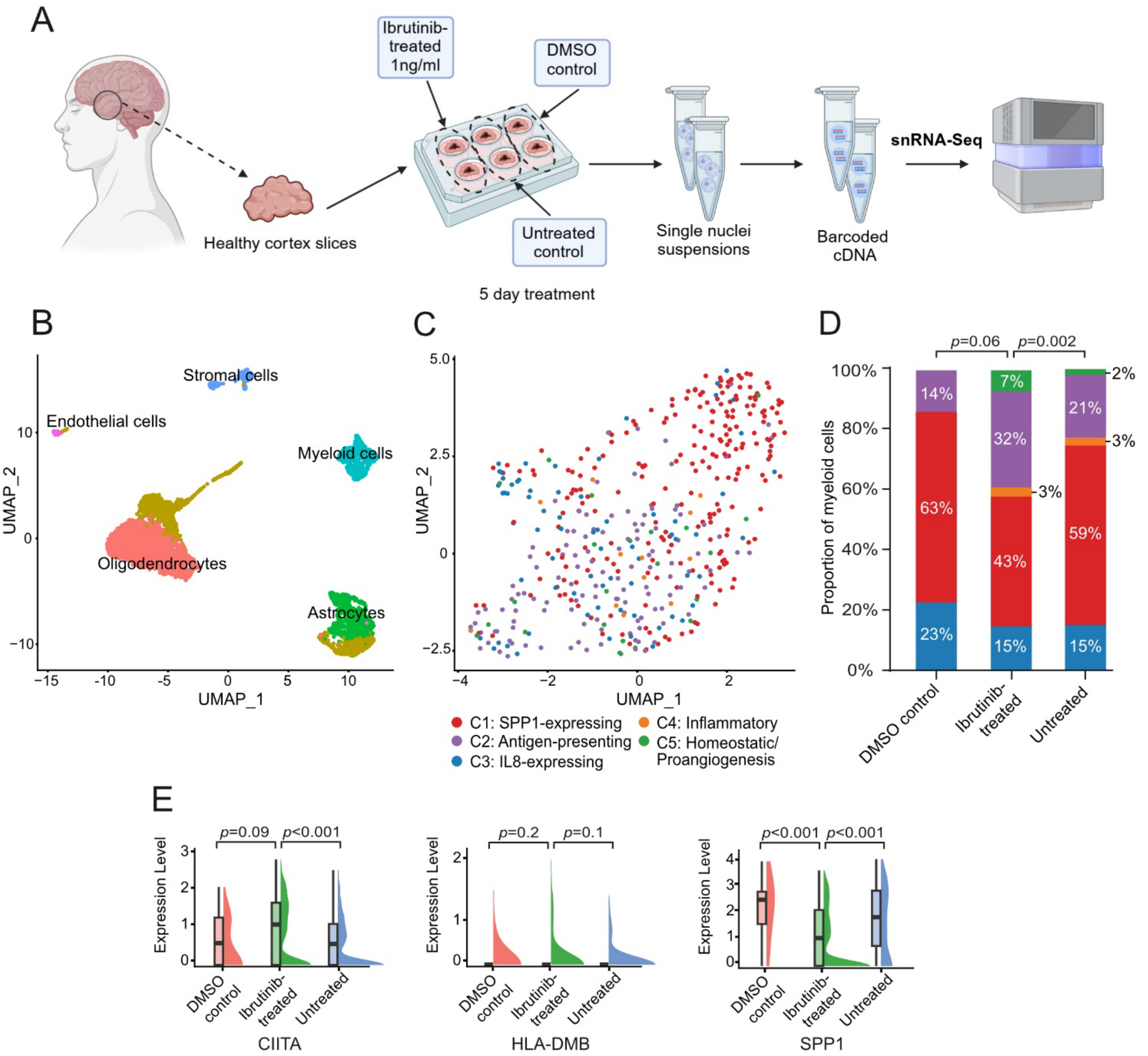

Supplementary Figure 5. Myeloid compartment of non-malignant cortical slices. (A) Non-malignant cortical slices were cultured and treated over five days either with ibrutinib solved in DMSO, with DMSO alone, or they remained untreated**.** snRNA-Seq was performed. **(B)** UMAP of the integrated snRNA-Seq dataset. Cells are colored by annotated clusters. **(C)** UMAP of the myeloid cell cluster of the snRNA-Seq dataset

from non-malignant cortical slices. Subclusters are colored. **(D)** The proportion of cells per subcluster to the total number of myeloid cells for each treatment condition is shown as a bar graph. **(E)** Rainbow plot of marker genes of the antigen-presenting myeloid subcluster and the SPP1 subcluster comparing the pseudo-bulk expression level of these genes among the three treatment conditions. Each plot shows the median, 25-75th percentile, and range of expression levels.

References

1. Hans CP, Weisenburger DD, Greiner TC, Gascoyne RD, Delabie J, Ott G, u. a. Confirmation of the molecular classification of diffuse large B-cell lymphoma by immunohistochemistry using a tissue microarray. Blood. 1. Januar 2004;103(1):275–82.

2. The WHO Classification of Tumours Editorial Board, Herausgeber. Haematolymphoid Tumours: Who Classification of Tumours. 5th ed. Lyon: International Agency for Research on Cancer; 2024. 1 S.

3. Ravi VM, Joseph K, Wurm J, Behringer S, Garrelfs N, d'Errico P, u. a. Human organotypic brain slice culture: a novel framework for environmental research in neuro-oncology. Life Sci Alliance. August 2019;2(4):e201900305.

4. Ravi VM, Neidert N, Will P, Joseph K, Maier JP, Kückelhaus J, u. a. T-cell dysfunction in the glioblastoma microenvironment is mediated by myeloid cells releasing interleukin-10. Nat Commun. 17. Februar 2022;13(1):925.

5. Orringer DA, Pandian B, Niknafs YS, Hollon TC, Boyle J, Lewis S, u. a. Rapid intraoperative histology of unprocessed surgical specimens via fibre-laser-based stimulated Raman scattering microscopy. Nat Biomed Eng. 2017;1:0027.

6. Yu H, Kong H, Li C, Dong X, Wu Y, Zhuang Y, u. a. Bruton's tyrosine kinase inhibitors in primary central nervous system lymphoma-evaluation of anti-tumor efficacy and brain distribution. Transl Cancer Res. Mai 2021;10(5):1975–83.

7. Mason C, Savona S, Rini JN, Castillo JJ, Xu L, Hunter ZR, u. a. Ibrutinib penetrates the blood brain barrier and shows efficacy in the therapy of Bing Neel syndrome. Br J Haematol. Oktober 2017;179(2):339–41.

8. Bernard S, Goldwirt L, Amorim S, Brice P, Brière J, de Kerviler E, u. a. Activity of ibrutinib in mantle cell lymphoma patients with central nervous system relapse. Blood. 1. Oktober 2015;126(14):1695–8.

9. Touil H, Roostaei T, Calini D, Diaconu C, Epstein S, Raposo C, u. a. A structured evaluation of cryopreservation in generating single-cell transcriptomes from cerebrospinal fluid. Cell Rep Methods. 24. Juli 2023;3(7):100533.

10. Korsunsky I, Millard N, Fan J, Slowikowski K, Zhang F, Wei K, u. a. Fast, sensitive and accurate integration of single-cell data with Harmony. Nat Methods. Dezember 2019;16(12):1289–96.

11. Aran D, Looney AP, Liu L, Wu E, Fong V, Hsu A, u. a. Reference-based analysis of lung single-cell sequencing reveals a transitional profibrotic macrophage. Nat Immunol. Februar 2019;20(2):163–72.

12. Mabbott NA, Baillie JK, Brown H, Freeman TC, Hume DA. An expression atlas of human primary cells: inference of gene function from coexpression networks. BMC Genomics. 20. September 2013;14:632.

13. Reddy A, Zhang J, Davis NS, Moffitt AB, Love CL, Waldrop A, u. a. Genetic and Functional Drivers of Diffuse Large B Cell Lymphoma. Cell. 5. Oktober 2017;171(2):481-494.e15.

14. Satija R, Farrell JA, Gennert D, Schier AF, Regev A. Spatial reconstruction of single-cell gene expression data. Nat Biotechnol. Mai 2015;33(5):495–502.

15. Patel AA, Zhang Y, Fullerton JN, Boelen L, Rongvaux A, Maini AA, u. a. The fate and lifespan of human monocyte subsets in steady state and systemic inflammation. J Exp Med. 3. Juli 2017;214(7):1913–23.

16. Gosselin D, Skola D, Coufal NG, Holtman IR, Schlachetzki JCM, Sajti E, u. a. An environment-dependent transcriptional network specifies human microglia identity. Science. 23. Juni 2017;356(6344):eaal3222.

17. Trapnell C, Cacchiarelli D, Grimsby J, Pokharel P, Li S, Morse M, u. a. The dynamics and regulators of cell fate decisions are revealed by pseudotemporal ordering of single cells. Nat Biotechnol. April 2014;32(4):381–6.

18. Andreatta M, Carmona SJ. UCell: Robust and scalable single-cell gene signature scoring. Comput Struct Biotechnol J. 2021;19:3796–8.

19. Mutter JA, Alig SK, Esfahani MS, Lauer EM, Mitschke J, Kurtz DM, u. a. Circulating Tumor DNA Profiling for Detection, Risk Stratification, and Classification of Brain Lymphomas. J Clin Oncol Off J Am Soc Clin Oncol. 20. März 2023;41(9):1684–94.

20. Martin M. Cutadapt removes adapter sequences from high-throughput sequencing reads. EMBnet.journal. 2. Mai 2011;17(1):10.

21. Dobin A, Davis CA, Schlesinger F, Drenkow J, Zaleski C, Jha S, u. a. STAR: ultrafast universal RNA-seq aligner. Bioinforma Oxf Engl. 1. Januar 2013;29(1):15–21.

22. Liao Y, Smyth GK, Shi W. featureCounts: an efficient general purpose program for assigning sequence reads to genomic features. Bioinforma Oxf Engl. 1. April 2014;30(7):923–30.

23. Love MI, Huber W, Anders S. Moderated estimation of fold change and dispersion for RNA-seq data with DESeq2. Genome Biol. 2014;15(12):550.

24. Steen CB, Luca BA, Esfahani MS, Azizi A, Sworder BJ, Nabet BY, u. a. The landscape of tumor cell states and ecosystems in diffuse large B cell lymphoma. Cancer Cell. 11. Oktober 2021;39(10):1422-1437.e10.

25. Bolotin DA, Poslavsky S, Mitrophanov I, Shugay M, Mamedov IZ, Putintseva EV, u. a. MiXCR: software for comprehensive adaptive immunity profiling. Nat Methods. Mai 2015;12(5):380–1.

26. Wright GW, Huang DW, Phelan JD, Coulibaly ZA, Roulland S, Young RM, u. a. A Probabilistic Classification Tool for Genetic Subtypes of Diffuse Large B Cell Lymphoma with Therapeutic Implications. Cancer Cell. 13. April 2020;37(4):551-568.e14.

27. Adalsteinsson VA, Ha G, Freeman SS, Choudhury AD, Stover DG, Parsons HA, u. a. Scalable whole-exome sequencing of cell-free DNA reveals high concordance with metastatic tumors. Nat Commun. 6. November 2017;8(1):1324.

28. Poell JB, Mendeville M, Sie D, Brink A, Brakenhoff RH, Ylstra B. ACE: absolute copy number estimation from low-coverage whole-genome sequencing data. Bioinforma Oxf Engl. 15. August 2019;35(16):2847–9.
